# Supplementary material for: Causal Relationship between Aging and Anorexia Nervosa: A White-Matter-Microstructure-Mediated Mendelian Randomization Analysis
Source: Biomedicines. 2024 Aug 16;12(8):1874. doi: 10.3390/biomedicines12081874 (PMC11351342; doi:10.3390/biomedicines12081874)
Supplement: Supplementary file 1 [file biomedicines-12-01874-s001.zip › Supplementary Figure/Figure S4/Singe SNP plot of MR analysis for TL to ubm-b-1916.pdf]

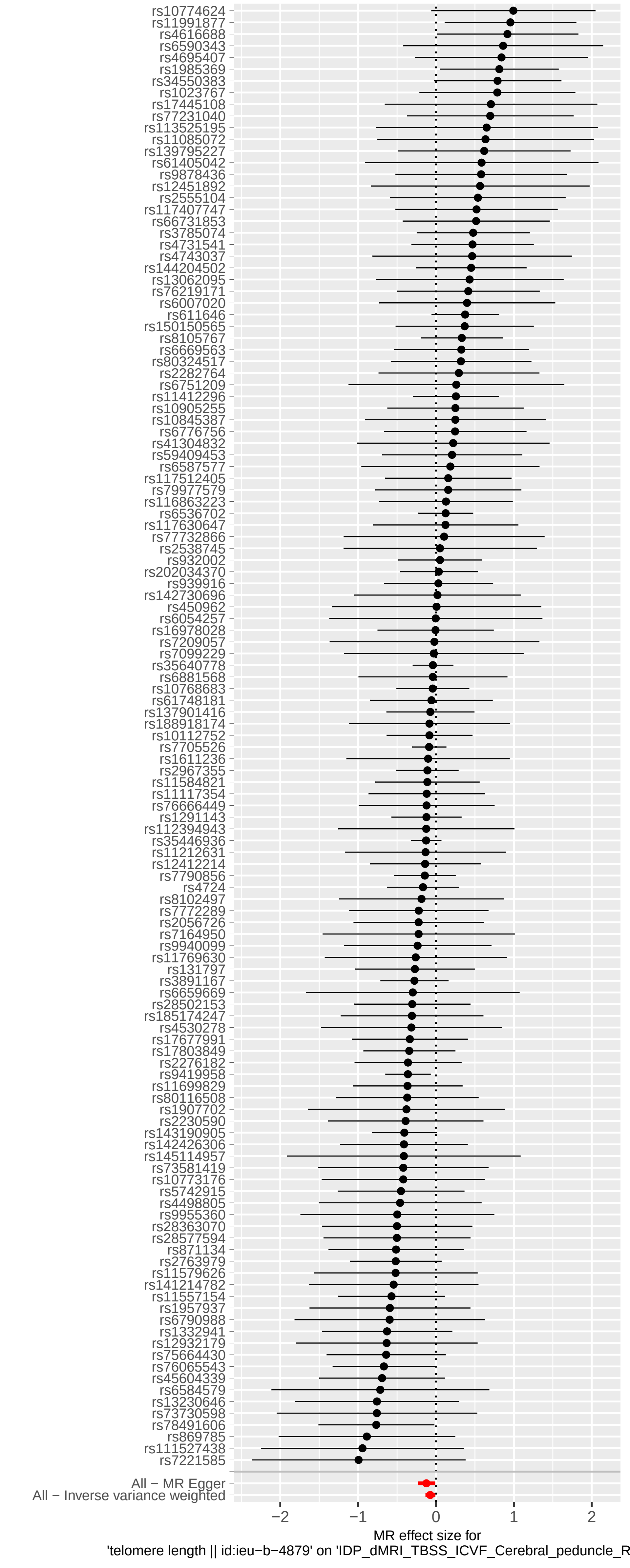
rs10774624
rs11991877
rs4616688
rs6590343
rs4695407
rs1985369
rs34550383
rs1023767
rs17445108
rs77231040
rs113525195
rs11085072
rs139795227
rs61405042
rs9878436
rs12451892
rs2555104
rs117407747
rs66731853
rs3785074
rs4731541
rs4743037
rs144204502
rs13062095
rs76219171
rs6007020
rs611646
rs150150565
rs8105767
rs6669563
rs80324517
rs2282764
rs6751209
rs11412296
rs10905255
rs10845387
rs6776756
rs41304832
rs59409453
rs6587577
rs117512405
rs79977579
rs116863223
rs6536702
rs117630647
rs77732866
rs2538745
rs932002
rs202034370
rs939916
rs142730696
rs450962
rs6054257
rs16978028
rs7209057
rs7099229
rs35640778
rs6881568
rs10768683
rs61748181
rs137901416
rs188918174
rs10112752
rs7705526
rs1611236
rs2967355
rs11584821
rs11117354
rs76666449
rs1291143
rs112394943
rs35446936
rs11212631
rs12412214
rs7790856
rs4724
rs8102497
rs7772289
rs2056726
rs7164950
rs9940099
rs11769630
rs131797
rs3891167
rs6659669
rs28502153
rs185174247
rs4530278
rs17677991
rs17803849
rs2276182
rs9419958
rs11699829
rs80116508
rs1907702
rs2230590
rs143190905
rs142426306
rs145114957
rs73581419
rs10773176
rs5742915
rs4498805
rs9955360
rs28363070
rs28577594
rs871134
rs2763979
rs11579626
rs141214782
rs11557154
rs1957937
rs6790988
rs1332941
rs12932179
rs75664430
rs76065543
rs45604339
rs6584579
rs13230646
rs73730598
rs78491606
rs869785
rs111527438
rs7221585
All − MR Egger
All − Inverse variance weighted
−2
−1
0
1
2
MR effect size for
'telomere length || id:ieu−b−4879' on 'IDP_dMRI_TBSS_ICVF_Cerebral_peduncle_R [illegible]
